# Phylogenetic profiles of all membrane transport proteins of the malaria parasite highlight new drug targets

**DOI:** 10.15698/mic2016.10.534

**Published:** 2016-08-30

**Authors:** January Weiner, Taco W.A. Kooij

**Affiliations:** 1 Department of Immunology, Max Planck Institute for Infection Biology, Berlin, Germany.; 2 Department of Medical Microbiology & Centre for Molecular and Biomolecular Informatics, Radboud Institute for Molecular Life Sciences, Radboud University Medical Centre, Nijmegen, The Netherlands.

**Keywords:** drug target, experimental genetics, malaria parasite, membrane transport protein, orthology, phylogeny, Plasmodium

## Abstract

In order to combat the on-going malaria epidemic, discovery of new drug targets remains vital. Proteins that are essential to survival and specific to malaria parasites are key candidates. To survive within host cells, the parasites need to acquire nutrients and dispose of waste products across multiple membranes. Additionally, like all eukaryotes, they must redistribute ions and organic molecules between their various internal membrane bound compartments. Membrane transport proteins mediate all of these processes and are considered important mediators of drug resistance as well as drug targets in their own right. Recently, using advanced experimental genetic approaches and streamlined life cycle profiling, we generated a large collection of *Plasmodium berghei* gene deletion mutants and assigned essential gene functions, highlighting potential targets for prophylactic, therapeutic, and transmission-blocking anti-malarial drugs. Here, we present a comprehensive orthology assignment of all *Plasmodium falciparum* putative membrane transport proteins and provide a detailed overview of the associated essential gene functions obtained through experimental genetics studies in human and murine model parasites. Furthermore, we discuss the phylogeny of selected potential drug targets identified in our functional screen. We extensively discuss the results in the context of the functional assignments obtained using gene targeting available to date.

## INTRODUCTION

The malaria parasite has adopted a highly complex life cycle involving a continuous switching between vertebrate hosts and anopheline mosquitoes. Within humans, *Plasmodium* species are obligate intracellular parasites moving through three different life-cycle stages. After an infectious mosquito bite, a single phase of preclinical growth within the host liver cells commences 1. Next, fast proliferating blood-stage parasites are the cause of malaria-associated pathology and severe disease outcome 2. Finally, some of the asexual blood-stage parasites are triggered to develop into male or female gametocytes, which are required for sexual reproduction following a mosquito blood meal 3,4.

Despite a gradual decline in annual malaria cases and deaths, the parasite remains one of the largest global killers. In 2015, WHO reported 150-300 million cases and 438,000 deaths 5. Difficulty in combating the disease is exacerbated by the growing resistance to anti-malarial drugs and the absence of an effective vaccine. The continuous need for new therapeutic anti-malarial drugs is unquestioned. Yet, due to limited availability, there is a much more pressing need for drugs that can prevent infections by acting prophylactically on the liver stage of the parasite, and transmission-blocking compounds, which kill the gametocytes thus helping to prevent the spread of the disease. Chemoprophylaxis is not only important for travellers 6 but also particularly for women in endemic areas in their first or second pregnancy 7. Thus, there is a critical need for novel drugs that may be used prophylactically, therapeutically, or to block transmission 8.

As defined by the Medicines for Malaria Venture in there 2015 annual report 9, ideal medicines for treatment and protection would both be suitable for mass drug administration programs and require a single encounter treatment, or better still a single exposure treatment, to help improve compliance. Treatment of infection should be effective against all life-cycle stages of all five malaria species infecting humans, including resistant strains, and resistance against the new drug should be difficult to achieve.

Generally, it is believed that these features may be best obtained by the combination of at least two active compounds, one fast acting for immediate clearance of the infection and a second, slower-acting compound providing long duration of efficacy. Furthermore, an ideal treatment would be gametocytocidal to prevent the spread of infection to mosquitoes, while sporontocidal or liver-stage activity would provide a prophylactic component. To complete the wish list of the ideal anti-malarial drug, it should also be active against the so-called hypnozoites, the dormant stages of certain malaria parasite species (most notably *P. vivax*) that are the cause of relapse infection even many years after the infectious mosquito bite. It is obvious that the development of the ideal anti-malarial drug will not be straightforward and will result in a compromise between the long list of desirable attributes and features. In this light, it is particularly important that on-going functional studies of malaria parasite biology continue to highlight potential new drug targets that have important roles in the different life-cycle stages of the parasite and are conserved among all malaria parasite species but are absent from or have diverged significantly in humans, such that compounds acting on these important *Plasmodium* proteins may do so effectively as well as selectively.

*Plasmodium* membrane transport proteins (MTP), such as the chloroquine resistance transporter (CRT) and the ATP-binding cassette (ABC) transporter family, including the multidrug resistance proteins (MDR) and the multidrug resistance-associated proteins (MRP), are well known for their roles in anti-malarial drug resistance 10,11. MTPs are also generally considered potential drug targets in their own right 12,13. Spiroindolones and dihydroisoquinolones are new classes of potent anti-malarial drugs, currently under clinical testing, that have both been shown to act via the *Plasmodium falciparum* cation ATPase, ATP4, causing severe disturbance of Na^+^ homeostasis in the parasite 14,15,16,17.

To survive and thrive, malaria parasites utilize a range of transport processes to import nutrients, export waste, and redistribute ions and small organic molecules between different sites and organelles 12,18. MTPs of different classes facilitate these processes. Following the functional and phylogenetic classification of MTPs from the Transporter Classification Database (http://www.tcdb.org 19, *Plasmodium* MTPs can be classified as: α-type channels and β-barrel porins (TCDB Class 1.A/B); P-P-bond hydrolysis-driven transporters, here referred to as pumps (TCDB Class 3.A); porters, including uniporters, symporters, and antiporters (TCDB Class 2.A); and unclassified, putative MTPs.

Experimental genetics is a powerful means to further explore critical functions of *Plasmodium* MTPs for parasite survival throughout its complex life cycle 20,21. Due to more efficient experimental and computational methods and access to the entire *in vivo* life cycle, the majority of such studies have been performed using murine malaria model species, notably *Plasmodium berghei*, and have focussed on single MTPs (Table S1). Two of our most recent studies have more than doubled the number of targeted genes, and have generated loss-of-function mutants in both *P. falciparum* and *P. berghei* 22,23. A systematic study of the MDR family demonstrated that four of seven members fulfil essential functions during blood-stage development, highlighting these as potential drug targets 22. While targeting 35 orphan MTPs, Kenthirapalan *et al.* produced the largest collection of *P. berghei* knock-out parasites available to date 23. The 29 available mutant lines provide a powerful resource for further studies of malaria parasite transport processes. In addition to highlighting six genes essential for blood-stage survival including five pumps, they also revealed potential prophylactic (MFS6) and transmission-blocking (ZIP1) drug targets 23.

Here, we performed comprehensive orthology profiling by reciprocal Blast of all identified 139 *Plasmodium* MTPs (consisting of the list published by Martin *et al.* 24 expanded with newly identified candidates) against a selection of 41 species from the entire breadth of the eukaryotic kingdom. To further validate the potential of the eight newly identified drug targets, we have explored their phylogenetic relationships in detail. These results are discussed extensively in the context of available insights from functional genetics studies of malaria parasite MTPs.

## RESULTS AND DISCUSSION

### Comprehensive orthology assignments of *Plasmodium* MTPs

To identify the levels of conservation of the 139 *P. falciparum* MTPs (Table S1) within the eukaryotic domain, we first performed an extensive orthology assignment using Blast against a set of 41 species (Table S2). We selected a subset of species to represent the enormous diversity in the eukaryotic domain by including sequences from all kingdoms, with an emphasis on protozoan species, commonly used model species, and parasite species of great medical or veterinary importance. These include the clinically second most important human malaria parasite *Plasmodium vivax* and the murine malaria model parasite *P. berghei*. *Plasmodium* species belong to a large monophyletic group of largely obligate intracellular parasites, the *Apicomplexa*. We included six additional apicomplexan parasites in our analysis: two piroplasms (*Babesia bovis* and *Theileria annulata*) that cause cattle fever and like *Plasmodium* species belong to the *Aconoidasida*, and four *Conoidasida*, including parasites of medical (*Toxoplasma gondii* and *Cryptosporidium parvum*) and veterinary (*Neospora caninum* and *Eimeria tenella*) importance.

Apicomplexan parasites are characterized by a complex at the apical end that is used to penetrate and enter a wide variety of host cells. However, this is not the only peculiar subcellular structure of these eukaryotic single cell parasites. Being eukaryotes, one would naturally expect most of the commonly shared organelles to be present, but while most do retain a single and thoroughly reduced mitochondrion, some have only vague remnants of the cell’s power house in the form of mitosomes 25. An additional organelle of endosymbiotic heritage present in the majority of apicomplexan parasites (though it has been lost in *Cryptosporidium* species) is the former-photosynthetic apicoplast 26. This plastid is of red algal origin, a trait shared across a variety of highly divergent species, including other alveolates, such as *Chromerida *and dinoflagellates, but not ciliates. For our analysis, we included four alveolates, including two ciliates and one of the closest relatives of the *Apicomplexa*, *Vitrella brassicaformis*.

The true evolutionary relationships between highly diverse protozoan lineages are unresolved and still a matter of dispute but many interesting parallels can be observed. Thus *Cryptomonads*, *Haptophyta*, and a significant proportion of *Stramenopiles*, all harbour plastids of red algal origin, although at present it is not clear whether these plastids originate from single or multiple secondary endosymobiotic events 27,28,29. In our analysis, we included one cryptomonad, *Guillardia theta*, as well as four stramenopiles including the oomycete *Phytophthora infestans*. The latter is a plant parasite causing potato blight, which does not harbour a plastid, but it does share another interesting feature with malaria parasites. Both oomycetes and malaria parasites extensively remodel the host they infect by exporting a large repertoire of effector proteins using comparable strategies 30. Further protist representative sequences were taken from two *Rhizaria*, six *Excavata*, including three kinetoplastid parasites, and two *Amoebozoa*.

From the plant kingdom, we took a green and a red alga and the most commonly used model plant *Arabidopsis thaliana*. Ophistokonts were represented by two fungi (baker’s yeast and the pathogenic *Cryptococcus neoformans*) and by nine animals, including the parasitic flatworm *Schistosoma haematobium*, the malaria mosquito *Anopheles gambiae*, a number of widely used model organisms, and of course mouse and human.

### Potential prophylactic drug targets

**Figure 1:**
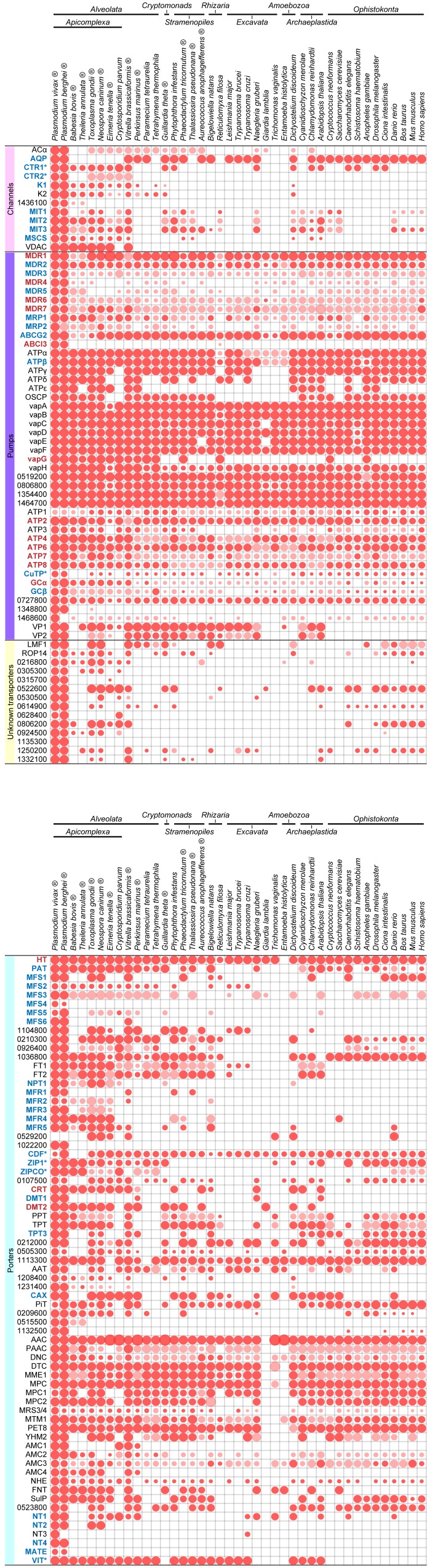
FIGURE 1: Orthologies of membrane transport proteins of the malaria parasite. Extensive amino acid based reciprocal homology searches were performed to establish orthologues of the all *Plasmodium* channels/pores (TCDB Class 1.A/B 19; pink), pumps (3.A; purple), porters (2.A; cyan), and unclassified, putative MTPs (yellow) in representative species of the entire breadth of the eukaryotic domain. Dot sizes indicate the fraction of the sequence length over which homology was detected; red dots indicate reciprocal orthologues. When a protein has not been given a name, these are indicated by their *P. falciparum* geneID number. MTPs for which the encoding genes were deleted successfully are indicated in blue, unsuccessful gene deletions suggesting essential functions during blood-stage development are highlighted in red. (®, species harbouring plastids of red algal origin; *, putative heavy metal transporting MTPs)

In our recent functional screen of orphan MTPs, we identified a critical role for a major facilitator superfamily member, *MFS6*, during liver-stage development, in addition to important functions in the blood 23. Though we identified a putative orthologue in *V. brassicaformis* (Figure 1), MFS6 is largely *Plasmodium*-specific and appears absent from humans. Its important functions during both liver- and blood-stage development justify further exploration of the protein as a target for compounds with combined prophylactic and therapeutic activity. *P. berghei* *MRP2*-deficient parasites demonstrate a complete arrest in the liver, a phenotype that was also observed in *P. falciparum* counterpart MRPs 31. Like MFS6, MRP2 appears to be largely *Plasmodium*-specific (Figure 1) providing another promising drug target. However, for these two MTPs both their function and their respective substrates remain unresolved.

Evidence is mounting that transport processes of metal ions, in particular of heavy metals such as iron, copper, and zinc, could provide efficient targets for chemoprophylactic treatments. Although the exact mechanisms are unclear, *P. berghei* liver-stage development is influenced by host iron homeostasis 32,33. Recently, a vacuolar iron transporter was described that plays an important (although not critical) role during liver infection *in vivo* and *in vitro* 34. Parasites lacking an alternative zinc-iron permease (ZIPCO) are severely affected in liver-stage development *in vitro* and show a delay in prepatency of two days 35. A similar delay in prepatency was observed following needle injection of *ctr2-* sporozoites despite developing normally in culture 23. Interestingly, these parasites were most severely affected during natural transmission by infectious mosquito bites or following subcutaneous injection of the sporozoites. Parasites deficient in the copper channel 1 gene (*CTR1*) completely failed to transmit, but this was at least in part attributable to a much reduced and delayed sporozoite production, and the infectivity of these sporozoites remains to be determined in more detail to establish whether CTR1 like CTR2 plays an important role in the liver 23. Despite the fact that the putative heavy metal transporting MTPs with a demonstrated important role during the establishment of new infection are not strictly essential, they may still prove interesting targets for prophylactic interventions, since none of the MTPs discussed above appear to have a clear reciprocal orthologue in humans and show only partial sequence matches (Figure 1).

### Potential transmission-blocking drug targets

The importance of heavy metal homeostasis appears not to be restricted to mosquito-to-mouse/human transition. Also sexual blood-stage parasites, parasite fertility, and effective colonization of the mosquito midgut appear to be strongly dependent on the correct distribution of these cations.

**Figure 2:**
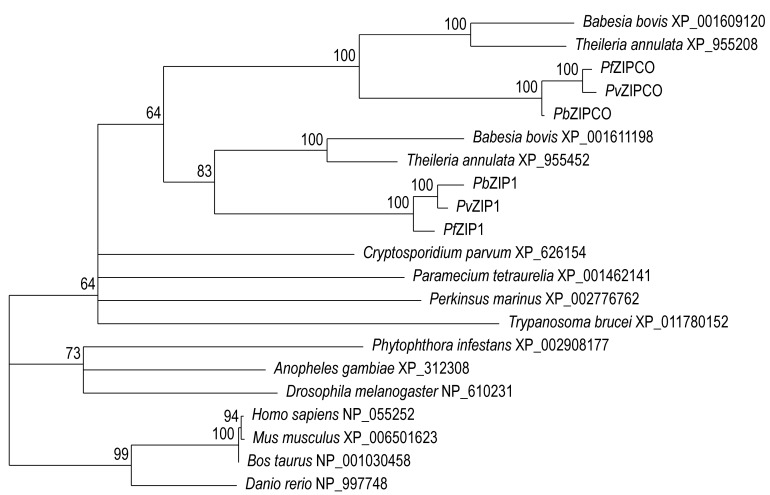
FIGURE 2: Phylogenetic tree iron-zinc permeases. ZIPCO and ZIP1 are two zinc-iron permeases, the first is important for mosquito-to-mouse transition, while the second is vital for male gametocyte formation and transmission to the malaria mosquito. This is a maximum likelihood tree of the orthologues found in a variety of eukaryotes, including humans. Nodes with support values <50 are left unresolved. The aconoidasidan paralogues ZIPCO and ZIP1 resolve with good support values, suggesting that the genes duplicated after differentiation of the *Aconoidasida*.

Where the two copper channels may act in copper transport in the liver stages, a copper-transporting P-type ATPase (CuTP) was shown to be central to fertility of both male and female gametes 36. Activation of *cutp-* male microgametes, a process known as exflagellation, is reduced to ~10% of wild-type and this reduction could be phenocopied using an intracellular copper chelator. Cross-fertilization studies and an even more pronounced reduction in oocyst numbers indicate additional important roles in the female gametes. ZIP1, a paralogue of the liver stage-specific ZIPCO, is crucial for mosquito colonization 23. Exflagellation in *zip1-* parasites is reduced to naught, which is directly attributable to a nearly complete absence of male gametocyte formation. Since *ZIP1*-deficient parasites also have a slightly reduced blood-stage multiplication rate, ZIP1 is potentially a very attractive transmission-blocking drug target. Indeed, if a compound would be able to target ZIPCO and ZIP1, this would be a triple-acting drug that could be used prophylactically, therapeutically, and to block spread of the disease. One disadvantage could be that humans harbour fourteen zinc-iron permeases 37, although only one of these bears any sequence similarity to the *Plasmodium* copies. Nevertheless, caution is needed to ensure that the compound is sufficiently parasite-specific. Phylogenetic profiling of the identified ZIP sequences supports a relatively recent gene duplication at the root of the *Aconoidasida* (Figure 2), and the relatively long distance from the vertebrate ZIPs suggest that specific targeting of the *Plasmodium* ZIPs might be feasible.

Other MTPs with roles during mouse/human-to-mosquito host transition have been identified, such as the Ca^2+^/H^+^ exchanger (CAX) 38, a cation diffusion factor or putative zinc transporter (CDF) 23, and the pantothenate transporter 23,39. Unfortunately, not a single MTP has been identified that is critical for both male and female gametocyte formation, although it remains to be tested if *zip1-* female gametocytes remain fertile or have lost the capacity to reproduce.

### Potential therapeutic drug targets

Two types of channels have been discussed as potential therapeutic drug targets. Two potassium channels (K1 and K2) were refractory to gene deletion in *P. falciparum* 40. However, absence of supporting evidence that this was not merely due to the technical difficulties of targeting *P. falciparum* genes and the fact that *Pb*K1 could be readily deleted 41 suggest that these channels may not be strictly necessary for blood-stage survival. The suitability of aquaglyceroporin (AQP) has also been subject of controversy. Initial studies demonstrated that blood-stage growth of *aqp-* parasites was strongly affected 42. Independently, we were only able to replicate a minor defect in Swiss-Webster mice 43. Using a sensitive flow cytometry - based method in NMRI mice, we saw no difference in growth rates of WT and *aqp-* parasites either growing in direct competition or in individual mice 44. The relevant difference between NMRI and Swiss-Webster mice that may lead to this observation is unclear.

Interestingly, a vast majority of the currently identified resistance markers, e.g. the ABC family 11, as well as the single validated druggable MTP, ATP4 16,17, are primary active transporters that require ATP to fuel their activity. As the parasite invests energy in their functioning, it is perhaps not surprising that many pumps play crucial roles at some stages during the parasite’s life cycle. Indeed, of all nineteen targeted pumps, eleven were shown to be refractory to gene deletion (Table S1). In addition to the four MDRs (MDR1, MDR4, MDR6, and MDR7) 22, these include the putative cation transporting ATPase, ATP4, (S. Kenthirapalan, K. Matuschewski, T.W.A. Kooij, unpublished data), ATP6 45 and the V-type proton ATPase subunit G 46, ABCI3, and four predicted aminophospholipid transporters 23.

Our orthology profiling indicates that the ABC transporters, with the exception of MDR1 (and to lesser extent MDR2), are poorly conserved across the eukaryotic kingdom, further highlighting their potential as anti-malarial drug targets (Figure 1). ABCI3 appears to be a unique, *Plasmodium*-specific ABC family member and is characterized by the presence of two transmembrane domains consisting of multiple transmembrane helices interspersed by a single nucleotide-binding domain. An extensive phylogenetic profiling of all ABC transporters identified in 16 eukaryotic and 55 prokaryotic genomes assigned *Pf*ABCI3 to a poorly supported clade together with sequences from two archaea from two different kingdoms and sequences from five bacteria from four different phyli 47. Thus, the orthology and phylogeny of ABCI3, along with demonstrated essential function during blood-stage development, strengthen its potential as a therapeutic drug target.

**Figure 3:**
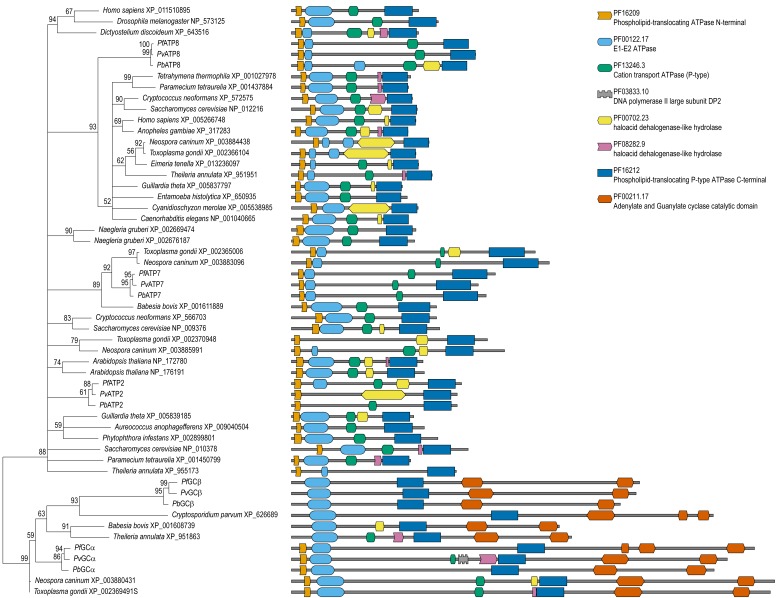
FIGURE 3: Phylogenetic tree of putative flippases. Four of five *Plasmodium berghei* putative aminophospholipid-transporting P_4_-type ATPases were shown to be essential for blood-stage development *in vivo*. This is a maximum likelihood tree based on the complete sequences of the identified orthologues along with models of all the domain architectures. Nodes with support values <50 are left unresolved. This unrooted tree shows low resolution at the base, making it difficult to interpret their phylogenetic history despite the fact that the proteins share their structures. ATP8 forms a well-supported clade with orthologues from a variety of species, while the apicomplexan branches with poor orthology assignments (GCα, GCβ, and ATP7) resolve well supporting the candidacy of GCα and ATP7 as therapeutic drug targets.

Another class of primary active transporters that was highlighted for its druggable potential consists of putative aminophospholipid-transporting P_4_-type ATPases, from hereon referred to as flippases 23. In addition to ATP2, ATP7, and ATP8, these also include two putatively bifunctional proteins that also harbour guanylyl cyclase (GC) activity 48. GCα, like the other flippases, was essential for blood-stage development, whereas GCβ plays a critical role in colonization of the mosquito midgut 23,49,50. While parts of the GCs are conserved, no bifunctional orthologues were found outside the apicomplexan clade (Figure 1). The phylogenetic tree of the flippases confirmed this notion (Figure 3). Orthology and phylogenetic profiles further indicate that ATP2 and ATP8 are rather well conserved including human orthologues, whereas ATP7 is largely apicomplexan-specific (Figure 1). Combined, these data suggest that ATP7 and GCα may form attractive targets for novel anti-malarial compounds. Of note, for one *P. falciparum* gene encoding a putative flippase (PF3D7_1468600), no orthologue exists in *P. berghei* and a possible essential role has not yet been established.

**Figure 4:**
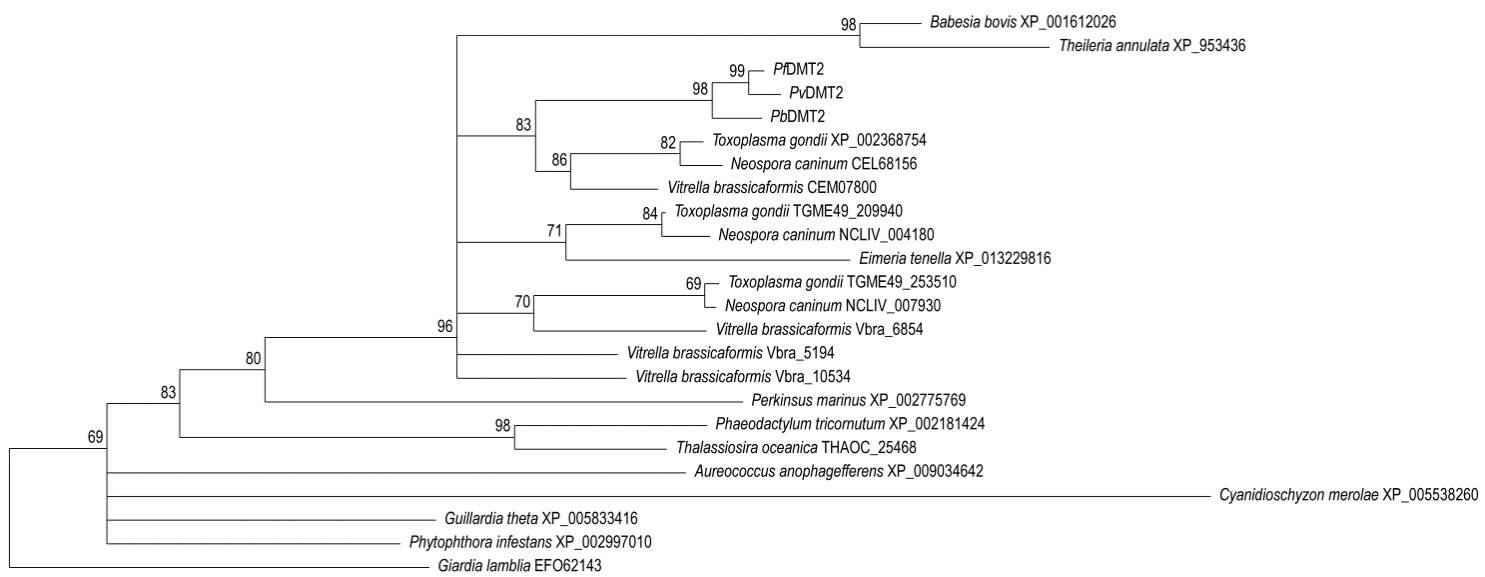
FIGURE 4: Phylogenetic tree of drug metabolite transporter 2 (DMT2). *Plasmodium berghei* DMT2 was shown to be essential for blood-stage development *in vivo*. This is a maximum likelihood tree of all homologues found in chromalveolate species and the red alga *Cyanidioschyzon merolae* using the *Giardia lamblia* homologue as an outgroup. Nodes with support values <50 are left unresolved. Apicomplexan DMT2 sequences, including the different paralogous sequences, form a distinct and well-supported clade, while the red algal distant homologue and the majority of other chromalveolates sequences sit unresolved at the base of this clade.

Of the largest group of MTPs, the porters (TCDB Class 2.A) 19, 28 genes have been targeted in *P. berghei*, only three times without success (Table S1). In addition to the well-studied CRT 51, these include a hexose transporter HT 52, which is well conserved including in humans (Figure 1), and the putative drug metabolite transporter DMT2 23. The latter presents a particularly interesting case considering the orthology discovery. Orthologues were identified in all apicomplexan parasites, with the exception of *Cryptosporidium*, in other alveolates, but not ciliates, and in the cryptomonad, *G. theta*, and most stramenopiles. Most species have a single *DMT2* but *T. gondii*, *N. caninum*, and* V. brassicaformis* have multiple copies. While the exact phylogenetic relationships of the alveolates, cryptomonads, and stramenopiles is still a topic of debate, many of these chromalveolates, as they are commonly referred to, harbour a plastid of red algal origin. Ciliates and *Cryptosporidium* species do not have such a plastid and thus it is tempting to speculate that DMT2 localizes to this endosymbiotic organelle. Indeed, the red alga *Cyanidioschizon merolae* appears to harbour a sequence with a very weak similarity that was initially just below the cut-off E-value applied to establish significant homologies. However, the presence of a rather well conserved copy in *P. infestans*, that lacks a relic plastid, and presence of weak similarities in three other unrelated eukaryotes (*Giardia lamblia*, *Dictyostelium discoideum*, and *Chlamydomonas reinhardtii*) suggest that the evolutionary history of this gene may be more complicated. When building a maximum likelihood tree for all identified homologues, including the few hits from unrelated species, the tree does not resolve well (data not shown). However, when only including sequences from the chromalveolates and the red alga, the tree is well-supported showing phylum-specific clades with *Cyanidioschizon merolae* as the outmost group of the tree (data not shown). Attempts to include other sequences as outgroup, *e.g.* *Plasmodium* DMT1 sequences, were unsuccessful due to a lack in sequence similarity and the consequently poorly resolving sequence alignments. The most consistent results were obtained when using a single DMT2 homologue of one of the three unrelated species as an outgroup (Figure 4).

Despite the significant uncertainties and remaining unresolved questions about the origin of DMT2, these data could well suggest that this essential MTP is localized in the parasite’s apicoplast. Our initial localization studies were hampered by very low expression levels of the protein and hence difficult to interpret, but appeared to indicate an intraparasitic staining including a specific, small structure that may well be the apicoplast 23. In blood-stage parasites, the single critical role of the apicoplast was shown to be the production of isopentenyl pyrophosphate (IPP) 53 and it is tempting to speculate that DMT2 is the dedicated IPP transport protein. However, DMT2 may also be involved in critical processes for the maintenance of the organelle and the IPP biosynthesis pathway, e.g. through the import of sulphur or iron into the organelle for essential iron-sulphur cluster biosynthesis 54.

### Conclusions

In conclusion, our extensive orthology assignment and phylogenetic profiling, in combination with published experimental genetics studies, particularly in the murine malaria model parasite *P. berghei*, support the candidacy of a number of prophylactic, therapeutic, and transmission-blocking drug targets. Further studies into the biochemical and structural properties of these MTPs are required and deserve prioritization.

## MATERIALS AND METHODS

### Identification of putative orthologues

We first selected candidate protein sequences by homology search using Blast (*blastp*, version 2.2.29+) 55, against a set of pre-selected proteomes (Table S2) applying a threshold for being considered significant of E=5e^-4^. Next, for each candidate, we performed a reciprocal Blast search against the *Plasmodium falciparum* genome. To circumvent incomplete annotations of the genomes, we have additionally searched each query against the NCBI non-redundant (NR) database from March 19th, 2016. Hits on the same sequence were analysed and the overall coverage of the identified homology was calculated as the fraction of the query length. Candidates which returned the same protein as the original query (i.e., reciprocal blast hits) were kept as putative orthologues, while remaining hits were retained as unspecified homologues. We further inspected the putative homologues using the RADS algorithm 56 and the Needleman-Wunsch global alignment algorithm.

### Phylogenetic reconstruction

Groups of putative orthologues for selected proteins were aligned using the programme Clustal Omega v. 1.2.1 57 with default parameters. The alignments were then manually inspected and regions of low coverage and poor conservation were masked. Maximum likelihood phylogeny reconstruction was performed with the TreePuzzle 58 with 10,000 iterations and a mixed model of rate homogeneity.

### Domain analysis

Domains of protein sequences were detected with *hmmscan* from the HMMer package v. 3.1b1 (http://hmmer.org) and the HMM profile collection from the PfamA database (October 2015). Significant overlaps were solved by E-value precedence. Domain architectures were analysed in combination with phylogenetic trees using the DoMosaic programme 59.

## SUPPLEMENTAL MATERIAL

Click here for supplemental data file.

All supplemental data for this article are also available online at http://microbialcell.com/researcharticles/phylogenetic-profiles-of-all-membrane-transport-proteins-of-the-malaria-parasite-highlight-new-drug-targets/.

## References

[1] Lindner SE, Miller JL, Kappe SHI (2012). Malaria parasite pre-erythrocytic infection: preparation meets
opportunity.. Cell Microbiol.

[2] Miller LH, Ackerman HC, Su X-Z, Wellems TE (2013). Malaria biology and disease pathogenesis: insights for new
treatments.. Nat Med.

[3] Josling GA, Llinás M (2015). Sexual development in Plasmodium parasites: knowing when it's
time to commit.. Nat Rev Microbiol.

[4] Kooij TWA, Matuschewski K (2007). Triggers and tricks of Plasmodium sexual
development.. Curr Opin Microbiol.

[5] World Health Organization (2015). World malaria report 2015..

[6] Schlagenhauf P, Petersen E (2008). Malaria chemoprophylaxis: strategies for risk
groups.. Clin Microbiol Rev.

[7] Radeva-Petrova D, Kayentao K, Kuile ter FO, Sinclair D, Garner P (2014). Drugs for preventing malaria in pregnant women in endemic areas:
any drug regimen versus placebo or no treatment.. Cochrane Database Syst Rev.

[8] Wells TNC, van Huijsduijnen RH, Van Voorhis WC (2015). Malaria medicines: a glass half full?. Nat Rev Drug Discov.

[9] Medicines for Malaria Venture (2015). MMV annual report 2015..

[10] Ecker A, Lehane AM, Clain J, Fidock DA (2012). PfCRT and its role in antimalarial drug
resistance.. Trends Parasitol.

[11] Koenderink JB, Kavishe RA, Rijpma SR, Russel FGM (2010). The ABCs of multidrug resistance in malaria.. Trends Parasitol.

[12] Kirk K, Lehane AM (2014). Membrane transport in the malaria parasite and its host
erythrocyte.. Biochem J.

[13] Kirk K (2004). Channels and transporters as drug targets in the
Plasmodium-infected erythrocyte.. Acta Trop.

[14] Rottmann M, McNamara CW, Yeung BKS, Lee MCS, Zou B, Russell B, Seitz P, Plouffe DM, Dharia NV, Tan J, Cohen SB, Spencer KR, González-Páez GE, Lakshminarayana SB, Goh A, Suwanarusk R, Jegla T, Schmitt EK, Beck H-P, Brun R, Nosten F, Renia L, Dartois V, Keller TH, Fidock DA, Winzeler EA, Diagana TT (2010). Spiroindolones, a potent compound class for the treatment of
malaria.. Science.

[15] Yeung BKS, Zou B, Rottmann M, Lakshminarayana SB, Ang SH, Leong SY, Tan J, Wong J, Keller-Maerki S, Fischli C, Goh A, Schmitt EK, Krastel P, Francotte E, Kuhen K, Plouffe D, Henson K, Wagner T, Winzeler EA, Petersen F, Brun R, Dartois V, Diagana TT, Keller TH (2010). Spirotetrahydro β-carbolines (spiroindolones): a new class of
potent and orally efficacious compounds for the treatment of
malaria.. J Med Chem.

[16] Spillman NJ, Allen RJW, McNamara CW, Yeung BKS, Winzeler EA, Diagana TT, Kirk K (2013). Na+ regulation in the malaria parasite Plasmodium falciparum
involves the cation ATPase PfATP4 and is a target of the spiroindolone
antimalarials.. Cell Host Microbe.

[17] Jiménez-Díaz MB, Ebert D, Salinas Y, Pradhan A, Lehane AM, Myrand-Lapierre M-E, O'Loughlin KG, Shackleford DM, Justino de Almeida M, Carrillo AK, Clark JA, Dennis ASM, Diep J, Deng X, Duffy S, Endsley AN, Fedewa G, Guiguemde WA, Gómez MG, Holbrook G, Horst J, Kim CC, Liu J, Lee MCS, Matheny A, Martínez MS, Miller G, Rodríguez-Alejandre A, Sanz L, Sigal M, Spillman NJ, Stein PD, Wang Z, Zhu F, Waterson D, Knapp S, Shelat A, Avery VM, Fidock DA, Gamo F-J, Charman SA, Mirsalis JC, Ma H, Ferrer S, Kirk K, Angulo-Barturen I, Kyle DE, Derisi JL, Floyd DM, Guy RK (2014). (+)-SJ733, a clinical candidate for malaria that acts through
ATP4 to induce rapid host-mediated clearance of Plasmodium.. Proc Natl Acad Sci USA.

[18] Kirk K (2015). Ion regulation in the malaria parasite.. Annu Rev Microbiol.

[19] Saier MH, Reddy VS, Tamang DG, Västermark A (2014). The transporter classification database.. Nucleic Acids Res.

[20] Matz JM, Kooij TWA (2015). Towards genome-wide experimental genetics in the in vivo malaria
model parasite Plasmodium berghei.. Pathog Glob Health.

[21] de Koning-Ward TF, Gilson PR, Crabb BS (2015). Advances in molecular genetic systems in malaria.. Nat Rev Microbiol.

[22] Rijpma SR, van der Velden M, Annoura T, Matz JM, Kenthirapalan S, Kooij TWA, Matuschewski K, van Gemert G-J, van de Vegte-Bolmer M, Siebelink-Stoter R, Graumans W, Ramesar J, Klop O, Russel FGM, Sauerwein RW, Janse C, Franke-Fayard BMD, Koenderink JB (2016). Vital and dispensable roles of Plasmodium multidrug resistance
transporters during blood- and mosquito-stage development.. Mol Microbiol.

[23] Kenthirapalan S, Waters AP, Matuschewski K, Kooij TWA (2016). Functional profiles of orphan membrane transporters in the life
cycle of the malaria parasite.. Nat Commun.

[24] Martin RE, Ginsburg H, Kirk K (2009). Membrane transport proteins of the malaria
parasite.. Mol Microbiol.

[25] Vaidya AB, Mather MW (2009). Mitochondrial evolution and functions in malaria
parasites.. Annu Rev Microbiol.

[26] van Dooren GG, Striepen B (2013). The algal past and parasite present of the
apicoplast.. Annu Rev Microbiol.

[27] Archibald JM (2015). Endosymbiosis and Eukaryotic Cell Evolution.. Curr Biol.

[28] Zimorski V, Ku C, Martin WF, Gould SB (2014). Endosymbiotic theory for organelle origins.. Curr Opin Microbiol.

[29] Keeling PJ (2013). The number, speed, and impact of plastid endosymbioses in
eukaryotic evolution.. Annu Rev Plant Biol.

[30] Jiang RHY, Stahelin RV, Bhattacharjee S, Haldar K (2013). Eukaryotic virulence determinants utilize phosphoinositides at
the ER and host cell surface.. Trends Microbiol.

[31] Rijpma SR, van der Velden M, González-Pons M, Annoura T, van Schaijk BCL, van Gemert G-J, van den Heuvel JJMW, Ramesar J, Chevalley-Maurel S, Ploemen IHJ, Khan SM, Franetich J-F, Mazier D, de Wilt JHW, Serrano AE, Russel FGM, Janse C, Sauerwein RW, Koenderink JB, Franke-Fayard BMD (2016). Multidrug ATP-binding cassette transporters are essential for
hepatic development of Plasmodium sporozoites.. Cell Microbiol.

[32] Portugal S, Carret C, Recker M, Armitage AE, Gonçalves LA, Epiphanio S, Sullivan D, Roy C, Newbold CI, Drakesmith H, Mota MM (2011). Host-mediated regulation of superinfection in
malaria.. Nat Med.

[33] Ferrer P, Castillo-Neyra R, Roy CN, Sullivan DJ (2016). Dynamic control of hepatic Plasmodium numbers by hepcidin despite
elevated liver iron during iron supplementation.. Microbes Infect.

[34] Slavic K, Krishna S, Lahree A, Bouyer G, Hanson KK, Vera I, Pittman JK, Staines HM, Mota MM (2016). A vacuolar iron-transporter homologue acts as a detoxifier in
Plasmodium.. Nat Commun.

[35] Sahu T, Boisson B, Lacroix C, Bischoff E, Richier Q, Formaglio P, Thiberge S, Dobrescu I, Ménard R, Baldacci P (2014). ZIPCO, a putative metal ion transporter, is crucial for
Plasmodium liver-stage development.. EMBO Mol Med.

[36] Kenthirapalan S, Waters AP, Matuschewski K, Kooij TWA (2014). Copper-transporting ATPase is important for malaria parasite
fertility.. Mol Microbiol.

[37] Kambe T, Hashimoto A, Fujimoto S (2014). Current understanding of ZIP and ZnT zinc transporters in human
health and diseases.. Cell Mol Life Sci.

[38] Guttery DS, Pittman JK, Frénal K, Poulin B, McFarlane LR, Slavic K, Wheatley SP, Soldati-Favre D, Krishna S, Tewari R, Staines HM (2013). The Plasmodium berghei Ca2+/H+ exchanger, PbCAX, is essential for
tolerance to environmental Ca2+ during sexual development.. PLoS Pathog.

[39] Hart RJ, Lawres L, Fritzen E, Ben Mamoun C, Aly ASI (2014). Plasmodium yoelii vitamin B5 pantothenate transporter candidate
is essential for parasite transmission to the mosquito.. Sci Rep.

[40] Waller KL, McBride SM, Kim K, McDonald TV (2008). Characterization of two putative potassium channels in Plasmodium
falciparum.. Malar J.

[41] Ellekvist P, Maciel J, Mlambo G, Ricke CH, Colding H, Klaerke DA, Kumar N (2008). Critical role of a K+ channel in Plasmodium berghei transmission
revealed by targeted gene disruption.. Proc Natl Acad Sci USA.

[42] Promeneur D, Liu Y, Maciel J, Agre P, King LS, Kumar N (2007). Aquaglyceroporin PbAQP during intraerythrocytic development of
the malaria parasite Plasmodium berghei.. Proc Natl Acad Sci USA.

[43] Kenthirapalan S, Waters AP, Matuschewski K, Kooij TWA (2012). Flow cytometry-assisted rapid isolation of recombinant Plasmodium
berghei parasites exemplified by functional analysis of
aquaglyceroporin.. Int J Parasitol.

[44] Matz JM, Matuschewski K, Kooij TWA (2013). Two putative protein export regulators promote Plasmodium blood
stage development in vivo.. Mol Biochem Parasitol.

[45] Pulcini S, Staines HM, Pittman JK, Slavic K, Doerig C, Halbert J, Tewari R, Shah F, Avery MA, Haynes RK, Krishna S (2013). Expression in yeast links field polymorphisms in PfATP6 to in
vitro artemisinin resistance and identifies new inhibitor
classes.. J Infect Dis.

[46] Fonager J, Pasini EM, Braks JAM, Klop O, Ramesar J, Remarque EJ, Vroegrijk IOCM, van Duinen SG, Thomas AW, Khan SM, Mann M, Kocken CHM, Janse C, Franke-Fayard BMD (2012). Reduced CD36-dependent tissue sequestration of
Plasmodium-infected erythrocytes is detrimental to malaria parasite growth
in vivo.. J Exp Med.

[47] Xiong J, Feng J, Yuan D, Zhou J, Miao W (2015). Tracing the structural evolution of eukaryotic ATP binding
cassette transporter superfamily.. Sci Rep.

[48] Carucci DJ, Witney AA, Muhia DK, Warhurst DC, Schaap P, Meima M, Li JL, Taylor MC, Kelly JM, Baker DA (2000). Guanylyl cyclase activity associated with putative bifunctional
integral membrane proteins in Plasmodium falciparum.. J Biol Chem.

[49] Moon RW, Taylor CJ, Bex C, Schepers R, Goulding D, Janse C, Waters AP, Baker DA, Billker O (2009). A cyclic GMP signalling module that regulates gliding motility in
a malaria parasite.. PLoS Pathog.

[50] Hirai M, Arai M, Kawai S, Matsuoka H (2006). PbGCβ is essential for Plasmodium ookinete motility to invade
midgut cell and for successful completion of parasite life cycle in
mosquitoes.. J Biochem.

[51] Ecker A, Lakshmanan V, Sinnis P, Coppens I, Fidock DA (2011). Evidence that mutant PfCRT facilitates the transmission to
mosquitoes of chloroquine-treated Plasmodium gametocytes.. J Infect Dis.

[52] Slavic K, Straschil U, Reininger L, Doerig C, Morin C, Tewari R, Krishna S (2010). Life cycle studies of the hexose transporter of Plasmodium
species and genetic validation of their essentiality.. Mol Microbiol.

[53] Yeh E, Derisi JL (2011). Chemical rescue of malaria parasites lacking an apicoplast
defines organelle function in blood-stage Plasmodium
falciparum.. PLoS Biol.

[54] Haussig JM, Matuschewski K, Kooij TWA (2014). Identification of vital and dispensable sulfur utilization
factors in the Plasmodium apicoplast.. PLoS ONE.

[55] Camacho C, Coulouris G, Avagyan V, Ma N, Papadopoulos J, Bealer K, Madden TL (2009). BLAST+: architecture and applications.. BMC Bioinformatics.

[56] Terrapon N, Weiner J, Grath S, Moore AD, Bornberg-Bauer E (2014). Rapid similarity search of proteins using alignments of domain
arrangements.. Bioinformatics.

[57] Sievers F, Wilm A, Dineen D, Gibson TJ, Karplus K, Li W, Lopez R, McWilliam H, Remmert M, Söding J, Thompson JD, Higgins DG (2011). Fast, scalable generation of high-quality protein multiple
sequence alignments using Clustal Omega.. Mol Syst Biol.

[58] Schmidt HA, Strimmer K, Vingron M, Haeseler von A (2002). TREE-PUZZLE: maximum likelihood phylogenetic analysis using
quartets and parallel computing.. Bioinformatics.

[59] Moore AD, Held A, Terrapon N, Weiner J, Bornberg-Bauer E (2014). DoMosaics: software for domain arrangement visualization and
domain-centric analysis of proteins.. Bioinformatics.

