# Supplementary material for: Phylogenetic profiles of all membrane transport proteins of the malaria parasite highlight new drug targets
Source: Microb Cell. 2016 Aug 30;3(10):511–21. doi: 10.15698/mic2016.10.534 (PMC5348985; doi:10.15698/mic2016.10.534)
Supplement: Supplementary file 1 [file mic-03-511-s01.pdf]

Supplementary Information for:

Phylogenetic profiles of all membrane transport proteins of the malaria parasite highlight new drug targets

January Weiner 3rd and Taco W.A. Kooij

Content:

- **Supplementary Table S1:** Gene targeting of *Plasmodium* channels (pink), pumps (purple), porters (cyan), and putative MTPs (yellow).
- **Supplementary Table S2:** Pre-selected proteomes used for orthology searches.

TABLE S1. Gene targeting of *Plasmodium* channels/pores (pink), pumps (purple), porters (cyan), and putative MTPs (yellow).

Name	*Pf* GeneID	KØ	KI	*Pb* GeneID	KØ	KI	TC	*Pf* Product Description	References
**ACα**	PF3D7_1404600.1			PBANKA_1037500	√		1.A	adenylyl cyclase alpha	[60]
**AQP**	PF3D7_1132800			PBANKA_0915600	√	√	1.A.8	aquaglyceroporin	[42,43]
**CTR1**	PF3D7_1439000			PBANKA_1302900	√		1.A.56	copper transporter	[23]
**CTR2**	PF3D7_1421900			PBANKA_1021500	√		1.A.56	copper transporter, putative	[23]
**K1**	PF3D7_1227200	X		PBANKA_1442000	√		1.A.1	potassium channel	[40,41]
**K2**	PF3D7_1465500	X		PBANKA_1328900			1.A.1	potassium channel	[40]
	PF3D7_1436100			PBANKA_1008700			1.A.1	conserved Plasmodium membrane protein, unknown function	
**MIT1**	PF3D7_1120300			PBANKA_0927900	√		1.A.35	magnesium transporter, putative	[23]
**MIT2**	PF3D7_1304200.1			PBANKA_1402700	√		1.A.35	CorA-like Mg2+ transporter protein, putative	[23]
**MIT3**	PF3D7_1427600			PBANKA_1017000	√		1.A.35	CorA-like Mg2+ transporter protein, putative	[23]
**MSCS**	PF3D7_1107900	√		PBANKA_0939000	√		1.A.23	mechanosensitive ion channel protein	*[23]
**VDAC**	PF3D7_1432100			PBANKA_1012800			1.B.8	voltage-dependent anion-selective channel protein, putative	
**MDR1**	PF3D7_0523000			PBANKA_1237800	X		3.A.1.201	multidrug resistance protein 1	[22]
**MDR2**	PF3D7_1447900	√		PBANKA_1311700	√	√	3.A.1.210	multidrug resistance protein 2	[22,23,61]
**MDR3**	PF3D7_1145500	√		PBANKA_0903500	√		3.A.1.209	ABC transporter B family member 3, putative	[22]
**MDR4**	PF3D7_0302600			PBANKA_0401200	X	√	3.A.1.209	ABC transporter B family member 4, putative	[22]
**MDR5**	PF3D7_1339900	√		PBANKA_1353300	√		3.A.1.201	ABC transporter B family member 5, putative	[22,61]
**MDR6**	PF3D7_1352100			PBANKA_1364800	X		3.A.1.210	ABC transporter B family member 6, putative	[22]
**MDR7**	PF3D7_1209900			PBANKA_0608300	X		3.A.1.209	ABC transporter B family member 7, putative	[22]
**MRP1**	PF3D7_0112200	√					3.A.1.208	multidrug resistance-associated protein 1	[31,62]
**MRP2**	PF3D7_1229100	√		PBANKA_1443800	√	√	3.A.1.208	multidrug resistance-associated protein 2	[31]
**ABCG2**	PF3D7_1426500	√	√	PBANKA_1018100	√		3.A.1.204	ABC transporter G family member 2	[23,63]
**ABCI3**	PF3D7_0319700			PBANKA_1218800	X	√	3.A.1	ABC transporter I family member 1, putative	[23]
**ATPα**	PF3D7_0217100			PBANKA_0313800			3.A.2	ATP synthase F1, alpha subunit	
**ATPβ**	PF3D7_1235700			PBANKA_1450300	√		3.A.2	ATP synthase subunit beta, mitochondrial	[64]
**ATPγ**	PF3D7_1311300			PBANKA_1409800			3.A.2	ATP synthase subunit gamma, mitochondrial	
**ATPδ**	PF3D7_1147700			PBANKA_0901400			3.A.2	mitochondrial ATP synthase delta subunit, putative	
**ATPε**	PF3D7_0715500			PBANKA_1421800			3.A.2	mitochondrial ATP synthase F1, epsilon subunit, putative	
**OSCP**	PF3D7_1310000			PBANKA_1408500			3.A.2	mitochondrial ATP synthase delta subunit, putative	
**vapA**	PF3D7_1311900			PBANKA_1410400			3.A.2	V-type proton ATPase catalytic subunit A	
**vapB**	PF3D7_0406100			PBANKA_1003800			3.A.2	V-type proton ATPase subunit B	
**vapC**	PF3D7_0106100			PBANKA_0207200			3.A.2	V-type proton ATPase subunit C, putative	
**vapD**	PF3D7_1341900			PBANKA_1355000			3.A.2	V-type proton ATPase subunit D, putative	
**vapE**	PF3D7_0934500			PBANKA_0835300			3.A.2	V-type proton ATPase subunit E, putative	
**vapF**	PF3D7_1140100			PBANKA_0908900			3.A.2	V-type proton ATPase subunit F, putative	
**vapG**	PF3D7_1323200			PBANKA_1338400	X		3.A.2	V-type proton ATPase subunit G, putative	[46]
**vapH**	PF3D7_1306600			PBANKA_1405100			3.A.2	V-type proton ATPase subunit H, putative	
	PF3D7_0519200			PBANKA_1234000			3.A.2	V-type proton ATPase 16 kDa proteolipid subunit	
	PF3D7_0806800			PBANKA_1223800			3.A.2	vacuolar proton translocating ATPase subunit A, putative	
	PF3D7_1354400			PBANKA_1131000			3.A.2	V-type proton ATPase 21 kDa proteolipid subunit, putative	
	PF3D7_1464700			PBANKA_1328100			3.A.2	ATP synthase (C/AC39) subunit, putative	
**ATP1**	PF3D7_0516100						3.A.3	cation-transporting ATPase 1	
**ATP2**	PF3D7_1219600			PBANKA_1434800	X	√	3.A.3	aminophospholipid-transporting P-ATPase	[23]
**ATP3**	PF3D7_0504000			PBANKA_1103600			3.A.3	cation transporting P-ATPase	
**ATP4**	PF3D7_1211900			PBANKA_0610400			3.A.3	non-SERCA-type Ca2+ -transporting P-ATPase	
**ATP6**	PF3D7_0106300	X	X	PBANKA_0207000	X	X	3.A.3	calcium-transporting ATPase	[45]
**ATP7**	PF3D7_0319000			PBANKA_0806300	X	√	3.A.3	P-type ATPase, putative	[23]
**ATP8**	PF3D7_1223400			PBANKA_1438300	X	√	3.A.3	phospholipid-transporting ATPase, putative	[23]
**CuTP**	PF3D7_0904900			PBANKA_0416500	√	√	3.A.3	copper-transporting ATPase	[36]
**GCα**	PF3D7_1138400			PBANKA_0910300	X	√	3.A.3	guanylyl cyclase	[23,49,50]
**GCβ**	PF3D7_1360500			PBANKA_1136700	√		3.A.3	guanylyl cyclase beta	[23,49,50]
	PF3D7_0727800			PBANKA_0211900			3.A.3	cation transporting ATPase, putative	
	PF3D7_1348800			PBANKA_1361600			3.A.3	E1-E2 ATPase, putative	
	PF3D7_1468600						3.A.3	aminophospholipid transporter, putative	
**VP1**	PF3D7_1456800			PBANKA_1449800			3.A.3.10	V-type H^+-translocating pyrophosphatase, putative	
**VP2**	PF3D7_1235200			PBANKA_1320500			3.A.3.10	V-type K^+-independent H^+-translocating inorganic pyrophosphatase	
**LMF1**	PF3D7_0824700			PBANKA_0705900			unknown	lipase maturation factor, putative	
**ROP14**	PF3D7_0613300			PBANKA_0111600			unknown	rhoptry protein ROP14	
	PF3D7_0216800			PBANKA_0313500			unknown	conserved Plasmodium membrane protein, unknown function	
	PF3D7_0305300			PBANKA_0403800			unknown	conserved Plasmodium membrane protein, unknown function	
	PF3D7_0315700			PBANKA_0413500			unknown	conserved Plasmodium membrane protein, unknown function	
	PF3D7_0522600			PBANKA_1237300			unknown	inner membrane complex protein	
	PF3D7_0530500			PBANKA_1244900			unknown	conserved Plasmodium membrane protein, unknown function	
	PF3D7_0614900			PBANKA_1229600			unknown	conserved Plasmodium membrane protein, unknown function	
	PF3D7_0628400			PBANKA_1127100			unknown	conserved Plasmodium membrane protein, unknown function	
	PF3D7_0806200			PBANKA_1224500			unknown	conserved Plasmodium membrane protein, unknown function	
	PF3D7_0924500			PBANKA_0825400			unknown	conserved Plasmodium membrane protein, unknown function	
	PF3D7_1135300			PBANKA_0913200			unknown	conserved Plasmodium membrane protein, unknown function	
	PF3D7_1250200			PBANKA_1463100			unknown	conserved Plasmodium membrane protein, unknown function	
	PF3D7_1332100			PBANKA_1346900			unknown	conserved Plasmodium membrane protein, unknown function	

TABLE S1 (cont.). Gene targeting of *Plasmodium* channels/pores (pink), pumps (purple), porters (cyan), and putative MTPs (yellow).

Name	*Pf* GeneID	KØ	KI	*Pb* GeneID	KØ	KI	TC	*Pf* Product Description	References
**HT**	PF3D7_0204700	X		PBANKA_0302500	X	√	2.A.1.1	hexose transporter	[23,39,52]
**PAT**	PF3D7_0206200			PBANKA_0303900	√		2.A.1.2	pantothenate transporter	
**MFS1**	PF3D7_0516500			PBANKA_1231300	√		2.A.1.2	metabolite/drug transporter, putative	[23]
**MFS2**	PF3D7_0916000			PBANKA_0817000	√		2.A.1.1	sugar transporter, putative	[23]
**MFS3**	PF3D7_0919500			PBANKA_0820400	√		2.A.1.1	sugar transporter, putative	[23]
**MFS4**	PF3D7_1203400			PBANKA_0602400	√		2.A.1	transporter, putative	[23]
**MFS5**	PF3D7_1428200			PBANKA_1016400	√		2.A.1.2	metabolite/drug transporter, putative	[23]
**MFS6**	PF3D7_1440800			PBANKA_1304700	√		2.A.1.2	major facilitator superfamily, putative	[23]
	PF3D7_1104800			PBANKA_0942100			2.A.1.2	metabolite/drug transporter, putative	
	PF3D7_0210300			PBANKA_0307200			2.A.1.13	monocarboxylate transporter, putative	
	PF3D7_0926400			PBANKA_0827200			2.A.1.13	monocarboxylate transporter, putative	
	PF3D7_1036800			PBANKA_0519800			2.A.1.25	acetyl-CoA transporter, putative	
**FT1**	PF3D7_0828600			PBANKA_0702100			2.A.71	folate transporter 1	
**FT2**	PF3D7_1116500			PBANKA_0931500			2.A.71	folate transporter 2	
**NPT1**	PF3D7_0104800			PBANKA_0208300	√	√	2.A.?	novel putative transporter 1	[23,65]
**MFR1**	PF3D7_0614300			PBANKA_0112500	√		2.A.60	organic anion transporter	[23]
**MFR2**	PF3D7_0104700			PBANKA_0208400	√		2.A.?	transporter, putative	[23]
**MFR3**	PF3D7_0312500			PBANKA_0410500	√		2.A.?	transporter, putative	[23]
**MFR4**	PF3D7_0914700			PBANKA_0815700	√		2.A.?	transporter, putative	[23]
**MFR5**	PF3D7_1129900			PBANKA_0918300	√		2.A.?	transporter, putative	[23]
	PF3D7_0529200						2.A.2	sugar transporter, putative	
	PF3D7_1022200			PBANKA_0506400			2.A.71	conserved Plasmodium membrane protein, unknown function	
**CDF**	PF3D7_0715900			PBANKA_1422200	√		2.A.4	zinc transporter, putative	[23]
**ZIP1**	PF3D7_0609100			PBANKA_0107700	√	√	2.A.5	Zn2+ or Fe2+ permease	[23,66]
**ZIPCO**	PF3D7_1022300			PBANKA_0506500	√	√	2.A.5	ZIP domain-containing protein, putative	[35]
	PF3D7_0107500			PBANKA_0205900			2.A.6.6	lipid/sterol:H+ symporter	
**CRT**	PF3D7_0709000	X		PBANKA_1219500	X		2.A.7.3	chloroquine resistance transporter	[51]
**DMT1**	PF3D7_0715800			PBANKA_1422100	√		2.A.7.3	drug/metabolite exporter, drug/metabolite transporter	[23]
**DMT2**	PF3D7_0716900			PBANKA_0614600	X	√	2.A.7	drug metabolite transporter, putative	[23]
**PPT**	PF3D7_0530200			PBANKA_1244600			2.A.7.9	phosphoenolpyruvate/phosphate translocator	
**TPT**	PF3D7_0508300			PBANKA_1107900			2.A.7.9	triose phosphate transporter	
**TPT3**	PF3D7_1218400			PBANKA_1434000	√		2.A.7.9	triose or hexose phosphate/phosphate translocator, putative	[23]
	PF3D7_0212000			PBANKA_0308800			2.A.7.13	GDP-fructose:GMP antiporter, putative	
	PF3D7_0505300			PBANKA_1104900			2.A.7.10	UDP-N-acetyl glucosamine:UMP antiporter	
	PF3D7_1113300			PBANKA_0934300			2.A.7.11	UDP-galactose transporter, putative	
**AAT**	PF3D7_0629500						2.A.18	amino acid transporter, putative	
	PF3D7_1208400			PBANKA_0606900			2.A.18	amino acid transporter, putative	
	PF3D7_1231400			PBANKA_1446100			2.A.18	amino acid transporter, putative	
**CAX**	PF3D7_0603500			PBANKA_0102300	√	√	2.A.19	cation/H+ antiporter	[38]
**PiT**	PF3D7_1340900			PBANKA_1354300			2.A.20	sodium-dependent phosphate transporter	
	PF3D7_0209600			PBANKA_0306700			2.A.22	transporter, putative	
	PF3D7_0515500			PBANKA_1115100			2.A.22	amino acid transporter, putative	
	PF3D7_1132500			PBANKA_0915900			2.A.22	amino acid transporter, putative	
**AAC**	PF3D7_1037300			PBANKA_0520200			2.A.29	ADP/ATP transporter on adenylate translocase	
**PAAC**	PF3D7_1004800			PBANKA_1203100			2.A.29	ADP/ATP carrier protein, putative	
**DNC**	PF3D7_1368700			PBANKA_1144600			2.A.29	deoxyribonucleotide carrier	
**DTC**	PF3D7_0823900			PBANKA_0706700			2.A.29	dicarboxylate/tricarboxylate carrier	
**MPC**	PF3D7_1202200			PBANKA_0601100			2.A.29	mitochondrial phosphate carrier protein	
**MPC1**	PF3D7_1340800			PBANKA_1354200			2.A.150.1	mitochondrial pyruvate carrier protein 1, putative	
**MPC2**	PF3D7_1470400			PBANKA_1333600			2.A.150.1	mitochondrial pyruvate carrier protein 2, putative	
**MRS3/4**	PF3D7_0905200			PBANKA_0416200			2.A.29	mitochondrial carrier protein, putative	
**MTM1**	PF3D7_0407500			PBANKA_1005100			2.A.29	mitochondrial carrier protein, putative	
**PET8**	PF3D7_1241600			PBANKA_1455000			2.A.29	mitochondrial carrier protein, putative	
**YHM2**	PF3D7_1223800			PBANKA_1438700			2.A.29	mitochondrial carrier protein, putative	
**MME1**	PF3D7_0108400.1			PBANKA_0205100			2.A.29	mitochondrial magnesium exporter	
**AMC1**	PF3D7_0108800			PBANKA_0204700			2.A.29	conserved Plasmodium protein, unknown function	
**AMC2**	PF3D7_0811100			PBANKA_1426400			2.A.29	mitochondrial carrier protein, putative	
**AMC3**	PF3D7_0908800			PBANKA_0810000			2.A.29	transporter, putative	
**AMC4**	PF3D7_1033000			PBANKA_0516800			2.A.?	conserved Plasmodium protein, unknown function	
**NHE**	PF3D7_1303500			PBANKA_1402000			2.A.36	sodium/hydrogen exchanger, Na+, H+ antiporter	
**FNT**	PF3D7_0316600			PBANKA_0414400			2.A.44	formate-nitrite transporter	
**SulP**	PF3D7_1471200			PBANKA_1334400			2.A.53	inorganic anion exchanger, inorganic anion antiporter	
	PF3D7_0523800			PBANKA_1238600			2.A.55	transporter, putative	
**NT1**	PF3D7_1347200	√		PBANKA_1360100	√		2.A.57	nucleoside transporter 1	[67-69]
**NT2**	PF3D7_0824400			PBANKA_0706200	√		2.A.57	nucleoside transporter 2	[23]
**NT3**	PF3D7_1469400						2.A.57	nucleoside transporter 3, putative	
**NT4**	PF3D7_0103200			PBANKA_0209900	√		2.A.57	nucleoside transporter 4	[23]
**MATE**	PF3D7_0212800			PBANKA_0309700	√		2.A.66.1	multidrug efflux pump, putative	[23]
**VIT**	PF3D7_1223700			PBANKA_1438600	√	√	2.A.89	vacuolar iron transporter, putative	[34]

KØ, gene deletion or disruption; KI, endogenous tagging confirming locus accessibility; TC, transporter classification [19]; X, refractory; √, mutant parasite line generated successfully; * Rayavara K, Desai SA (**2008**). Genetic disruption of a mechanosensitive ion channel in *Plasmodium falciparum*. **Am J Trop Med Hyg** 79(sup 6): 65-66.

TABLE S2. Pre-selected proteomes used for orthology searches (parasite sequences highlighted in blue and species with a plastid of red algal origin are marked with ®).

Species	Common name	ID	Source
Plasmodium falciparum ®	Malaria parasite	GCF_000002765.3_ASM276v1	Refseq/NCBI
Plasmodium vivax ®	Malaria parasite (tertian)	GCF_000002415.2_ASM241v2	Refseq/NCBI
Plasmodium berghei ®	Rodent malaria model parasite		WTSI [70]
Babesia bovis ®	Piroplasm parasite (babesiosis, cattle fever)	GCF_000165395.1_ASM16539v1	Refseq/NCBI
Theileria annulata ®	Piroplasm parasite (theileriosis, cattle fever)	GCF_000003225.2_ASM322v1	Refseq/NCBI
Toxoplasma gondii ®	Coccidian parasite (toxoplasmosis)	GCF_000006565.1_JCVI_tgg_v1.0	Refseq/NCBI
Neospora caninum ®	Coccidian parasite (neosporosis in canids & cattle)	GCF_000208865.1_ASM20886v2	Refseq/NCBI
Eimeria tenella ®	Coccidian parasite (cecal coccidiosis in poultry)	GCF_000499545.1_ETH001	Refseq/NCBI
Cryptosporidium parvum	Coccidian parasite (cryptosporidiosis)	GCF_000165345.1_ASM16534v1	Refseq/NCBI
Vitrella brassicaformis ®	Chromerid spp.	ccmp3155.GCA_001179505.1.30.pep.all	ENSEMBL
Perkinsus marinus ®	Perkinsid spp. (perkinsosis in oysters)	GCF_000006405.1_JCVI_PMG_1.0	Refseq/NCBI
Paramecium tetraurelia	Ciliate model	GCF_000165425.1_ASM16542v1	Refseq/NCBI
Tetrahymena thermophila	Ciliate model	GCF_000189635.1_JCVI-TTA1-2.2	Refseq/NCBI
Guillardia theta ®	Cryptomonad spp.	GCF_000315625.1_Guith1	Refseq/NCBI
Phytophthora infestans	Oomycete parasite (potato blight)	GCF_000142945.1_ASM14294v1	Refseq/NCBI
Phaeodactylum tricornutum ®	Diatom model	GCF_000150955.2_ASM15095v2	Refseq/NCBI
Thalassiosira pseudonana ®	Diatom model	GCF_000149405.2_ASM14940v2	Refseq/NCBI
Aureococcus anophagefferens ®	Heterokont alga spp.	GCF_000186865.1_v_1.0	Refseq/NCBI
Bigelowiella natans	Cercozoa model	Bigna1_filtered_proteins (17 Mar 2016)	JGI
Reticulomyxa filosa	Foraminifera model	GCA_000512085.1_Reti_assembly1.0	Refseq/NCBI
Leishmania major	Kinetoplastid parasite (leishmaniasis)	GCF_000002725.2_ASM272v2	Refseq/NCBI
Trypanosoma brucei	Kinetoplastid parasite (African sleeping sickness)	GCF_000210295.1_ASM21029v1	Refseq/NCBI
Trypanosoma cruzi	Kinetoplastid parasite (Chagas disease)	GCF_000209065.1_ASM20906v1	Refseq/NCBI
Naegleria gruberi	Non-parasitic excavata spp.	GCF_000004985.1_V1.0	Refseq/NCBI
Giardia lamblia	Flagellated protozoan parasite (giardiasis)	GCF_000002435.1_GL2	Refseq/NCBI
Trichomonas vaginalis	Flagellated protozoan parasite (trichomoniasis)	GCF_000002825.2_ASM282v1	Refseq/NCBI
Entamoeba histolytica	Protozoan parasite (amoebiasis)	GCF_000208925.1_JCVI_ESG2_1.0	Refseq/NCBI
Dictyostelium discoideum	Slime mold: amoeba model	GCF_000004695.1_dicty_2.7	Refseq/NCBI
Cyanidioschyzon merolae	Red alga model	GCF_000091205.1_ASM9120v1	Refseq/NCBI
Chlamydomonas reinhardtii	Green alga model	GCF_000002595.1_v3.0	Refseq/NCBI
Arabidopsis thaliana	Thale cress: plant model	GCF_000001735.3_TAIR10	Refseq/NCBI
Cryptococcus neoformans	Yeast parasite (cryptococcosis)	GCF_000091045.1_ASM9104v1	Refseq/NCBI
Saccharomyces cerevisiae	Baker's yeast: yeast model	GCF_000146045.2_R64	Refseq/NCBI
Schistosoma haematobium	Flatworm parasite (schistosomiasis)	GCF_000699445.1_SchHae_1.0	Refseq/NCBI
Caenorhabditis elegans	Roundworm model	GCF_000002985.6_WBcel235	Refseq/NCBI
Anopheles gambiae	"Malaria mosquito"	GCF_000005575.2_AgamP3	Refseq/NCBI
Drosophila melanogaster	Fruit fly: arthropod model	GCF_000001215.4_Release_6_plus_ISO1_MT	Refseq/NCBI
Ciona intestinalis	Vase tunicate: chordate model	GCF_000224145.2_KH	Refseq/NCBI
Danio rerio	Zebrafish: fish model	GCF_000002035.5_GRCz10	Refseq/NCBI
Bos taurus	Cattle	GCF_000003055.6_Bos_taurus_UMD_3.1.1	Refseq/NCBI
Mus musculus	Mouse: mammal model	GCF_000001635.24_GRCm38.p4	Refseq/NCBI
Homo sapiens	Human	GCF_000001405.32_GRCh38.p6	Refseq/NCBI

Supplemented references:

60. Ono T, Cabrita-Santos L, Leitao R, Bettiol E, Purcell LA, Diaz-Pulido O, Andrews LB, Tadakuma T, Bhanot P, Mota MM, and Rodriguez A (**2008**). Adenylyl cyclase alpha and cAMP signaling mediate Plasmodium sporozoite apical regulated exocytosis and hepatocyte infection. **PLoS Pathog** 4(2): e1000008. doi: 10.1371/journal.ppat.1000008.

61. van der Velden M, Rijpma SR, Russel FG, Sauerwein RW, and Koenderink JB (**2015**). *Pf*MDR2 and *Pf*MDR5 are dispensable for *Plasmodium falciparum* asexual parasite multiplication but change *in vitro* susceptibility to anti-malarial drugs. **Malar J** 14(1): 581. doi: 10.1186/s12936-015-0581-y.

62. Raj DK, Mu J, Jiang H, Kabat J, Singh S, Sullivan M, Fay MP, McCutchan TF, and Su X-Z (**2009**). Disruption of a *Plasmodium falciparum* multidrug resistance-associated protein (*Pf*MRP) alters its fitness and transport of antimalarial drugs and glutathione. **J Biol Chem** 284(12): 7687–7696. doi: 10.1074/jbc.M806944200.

63. Tran PN, Brown SHJ, Mitchell TW, Matuschewski K, McMillan PJ, Kirk K, Dixon MWA, and Maier AG (**2014**). A female gametocyte-specific ABC transporter plays a role in lipid metabolism in the malaria parasite. **Nat Commun** 5: 4773. doi: 10.1038/ncomms5773.

64. Sturm A, Mollard V, Cozijnsen A, Goodman CD, and McFadden GI (**2015**). Mitochondrial ATP synthase is dispensable in blood-stage *Plasmodium berghei* rodent malaria but essential in the mosquito phase. **Proc Natl Acad Sci USA** : 201423959. doi: 10.1073/pnas.1423959112.

65. Boisson B, Lacroix C, Bischoff E, Gueirard P, Bargieri DY, Franke-Fayard BMD, Janse C, Ménard R, and Baldacci P (**2011**). The novel putative transporter NPT1 plays a critical role in early stages of *Plasmodium berghei* sexual development. **Mol Microbiol** 81(5): 1343–1357. doi: 10.1111/j.1365-2958.2011.07767.x.

66. Guerreiro A, Deligianni E, Santos JM, Silva PA, Louis C, Pain A, Janse C, Franke-Fayard BMD, Carret CK, Siden-Kiamos I, and Mair GR (**2014**). Genome-wide RIP-Chip analysis of translational repressor-bound mRNAs in the *Plasmodium* gametocyte. **Genome Biol** 15(11): 493. doi: 10.1186/s13059-014-0493-0.

67. Bissati El K, Zufferey R, Witola WH, Carter NS, Ullman B, and Ben Mamoun C (**2006**). The plasma membrane permease *Pf*NT1 is essential for purine salvage in the human malaria parasite *Plasmodium falciparum*. **Proc Natl Acad Sci USA** 103(24): 9286–9291. doi: 10.1073/pnas.0602590103.

68. Aly ASI, Downie MJ, Mamoun CB, and Kappe SHI (**2010**). Subpatent infection with nucleoside transporter 1-deficient *Plasmodium* blood stage parasites confers sterile protection against lethal malaria in mice. **Cell Microbiol** 12(7): 930–938. doi: 10.1111/j.1462-5822.2010.01441.x.

69. Niikura M, Inoue S-I, Mineo S, Yamada Y, Kaneko I, Iwanaga S, Yuda M, and Kobayashi F (**2013**). Experimental cerebral malaria is suppressed by disruption of nucleoside transporter 1 but not purine nucleoside phosphorylase. **Biochem Biophys Res Commun** 432(3): 504–508. doi: 10.1016/j.bbrc.2013.02.004.

70. Otto TD, Böhme U, Jackson AP, Hunt M, Franke-Fayard BMD, Hoeijmakers WAM, Religa AA, Robertson L, Sanders M, Ogun SA, Cunningham D, Erhart A, Billker O, Khan SM, Stunnenberg HG, Langhorne J, Holder AA, Waters AP, Newbold CI, Pain A, Berriman M, and Janse C (**2014**). A comprehensive evaluation of rodent malaria parasite genomes and gene expression. **BMC Biol** 12(1): 86. doi: 10.1186/s12915-014-0086-0.
